# Adherence Predictors in Pregnant Women Living with HIV on Tenofovir Alafenamide and Tenofovir Disoproxil Fumarate

**Published:** 2022-07-02

**Authors:** Ahizechukwu C Eke

**Affiliations:** * Department of Gynecology & Obstetrics, Johns Hopkins University School of Medicine, 600 N Wolfe St, Baltimore, MD 2128, USA

**Keywords:** Medication adherence, Tenofovir Disoproxil Fumarate, Tenofovir Alafenamide, Antiretroviral drugs, HIV AIDS, Pregnancy

## Abstract

**Background:**

Medication adherence to antiretroviral medications is critical during pregnancy in women living with HIV (WLHIV) for multiple reasons. In this study, we report medication adherence to tenofovir alafenamide (TAF) compared to tenofovir disoproxil fumarate (TDF) during pregnancy in WLHIV.

**Methods:**

This is a retrospective cohort study of pregnant women living with HIV aged 18-48 years who received either tenofovir alafenamide (TAF) or tenofovir disoproxil fumarate (TDF) during pregnancy. Medication adherence was assessed during each visit in all trimesters of pregnancy, and was self-reported. Demographics and outcomes were analyzed using standard statistical tests. Logistic regression analysis models accounting for potential confounders, with adjusted odds-ratios (aORs) and associated 95% confidence intervals were reported.

**Results:**

One hundred women met inclusion criteria, with thirty-four women on TAF and sixty-six women on TDF. While medication adherence was higher in women using TAF compared to TDF, with 76% adherent to TDF vs 83% adherent to TAF; p=0.282, in the 1^st^ trimester; 82% adherent to TDF vs 88% adherent to TAF; p=0.924, in the 2^nd^ trimester, and 88% adherent to TDF vs 91% adherent to TAF; p=0.176, in the 3^rd^ trimester of pregnancy, these differences in medication adherence were not statistically significant. In the third trimester of pregnancy, multiparous women were more likely to be adherent to TDF/TAF antiretroviral medications compared to nulliparous women - univariable odds ratio, OR 1.31, 95% CI 1.12, 1.57; p<0.05; multivariable (adjusted odds ratio, aOR 1.23, 95% CI 1.08, 1.52; p<0.05).

**Conclusions:**

Pregnant women living with HIV on TDF and TAF achieved high adherence, but medication adherence was better in the third trimester compared to the first or second trimesters of pregnancy. These findings support the need to continually assess medication adherence during pregnancy.

## INTRODUCTION

Human immunodeficiency infection during pregnancy continues to be a major cause of maternal and neonatal morbidity, especially in developing countries [1–3]. In 2020, approximately 1.3 million women living with HIV became pregnant, among which 85% (1.1 million) received antiretroviral therapy during pregnancy, according to the World Health Organization (WHO) [4]. Despite efforts to develop safe and effective HIV medications, pregnant and postpartum women remain one of the last groups (therapeutic orphans) to use newer HIV therapeutics, because drug development programs routinely exclude pregnant women [5]. Therefore, providing effective antiretroviral medications, efficiently, at the right doses, whilst measuring and supporting adherence and minimizing undesirable adverse effects, is essential to optimization efforts of antiretroviral therapy during pregnancy.

The introduction of highly active antiretroviral therapy (HAART) as a safe and effective method for treatment of HIV infection in high-risk populations changed the trajectory of survival from HIV infection, as well as perinatal HIV infections. Tenofovir disoproxil fumarate (TDF) and Tenofovir alafenamide (TAF) are two commonly used nucleoside reverse transcriptase inhibitors for preventing perinatal transmission [6–9]. Tenofovir alafenamide is a newer tenofovir (TFV) prodrug that is increasingly being used by pregnant women living with HIV (WLHIV) [9,10]. TAF achieves very high levels of tenofovir-diphosphate (TFV-DP) in peripheral blood mononuclear cells (PBMCs), and approximately 90% lower plasma concentrations of TFV compared to tenofovir disoproxil fumarate (TDF) [11,12]. The ability of TAF to selectively concentrate in leucocytes maximizes its antiviral efficacy and potency, making TAF more desirable when compared to TDF [12]. Several studies have compared the efficacy and safety of TDF to TAF in non-pregnant adults [13], but only very few epidemiologic studies of TAF use have been completed in pregnant women [6–9,14].

Medication adherence is a critical factor that impacts HIV progression during pregnancy [15,16]. While optimal adherence to antiretroviral therapy is crucial to preventing HIV perinatal transmission and viral resistance, sub-optimal adherence is common in pregnancy and the postpartum period [17,18]. Identifying pregnant and postpartum women at highest risk for poor adherence has become a major focus of current HIV research [19–21], and therefore, optimizing adherence among pregnant women living with HIV is critically important for the health of both the mother and the child [22]. As many women living with HIV are currently receiving TAF-based antiretroviral therapy, it is important to understand adherence to TAF, and what factors may modify HIV progression in adherent and non-adherent pregnant women living with HIV, and if TAF/TDF adherence is modified by any of these factors.

To facilitate understanding of adherence to tenofovir alafenamide and tenofovir disoproxil fumarate during pregnancy and postpartum, we conducted a retrospective cohort study among pregnant women living with HIV.

## METHODS

This medication adherence study was a retrospective cohort of pregnant women living with HIV managed at a single academic center - the Johns Hopkins Hospital antenatal HIV clinic during the periods that span from January 1^st^, 2015 and June 30^th^, 2020. The Johns Hopkins HIV antenatal outpatient clinic is the second largest provider of HIV/AIDS care during pregnancy in the state of Maryland, USA, and provides care to pregnant women living with HIV in the surrounding states of Virginia, West Virginia, Delaware, Pennsylvania, and Washington DC. Women met inclusion criteria if they were living with HIV, on a combination antiretroviral medication that included either a TDF or a TAF regimen, between the ages of 18 to 48 years of age, and greater than 5 weeks of gestation in the current pregnancy. Women were excluded if they were not living with HIV and not on a TDF or a TAF-based antiretroviral regimen. Over ninety-five percent of pregnant women living with HIV who attend the Johns Hopkins HIV in pregnancy antenatal clinic are on a TDF or a TAF containing regimen during pregnancy, and postpartum. Majority of pregnant women living with HIV were seen for their initial prenatal visit in the first trimester of pregnancy, and are continually seen until the time of delivery.

### Definition of exposure and outcome

Pregnant women living with HIV using combination antiretroviral therapy containing either TDF or TAF, was the primary exposure of interest. We defined each individual’s TAF or TDF adherence levels by trimesters of pregnancy (first, second and third trimesters of pregnancy). Combination antiretroviral therapy was defined as the combination of at least two nucleoside reverse transcriptase inhibitors and either a non-nucleoside reverse transcriptase inhibitor or a protease inhibitor, as per the U.S. Department of Health and Human Services recommendations [9]. The date of combined antiretroviral therapy initiation (either before the onset of pregnancy or once pregnancy was diagnosed), was documented in individual charts. Medication adherence to either a TDF or a TAF regimen was our primary outcome of interest.

### Covariates

For this study, several covariates of interest were measured at the beginning of pregnancy and at each trimester of pregnancy. Adherence was collected in two categories - optimal adherence (100%) and sub-optimal adherence (<100%), and therefore, we dichotomized adherence as such. Self-reported race was collected as: white non-Hispanic, white Hispanic, black non-Hispanic, black Hispanic, American Indian or Alaskan Native, Asian or Pacific Islander, other Hispanic and Other. Due to low numbers of racial ethnic group in our pregnant women, these categories were collapsed into two categories: Black and non-Blacks for the purpose of the analysis. For analysis purposes, parity was classified as nulliparous (no prior delivery), primiparous (one previous delivery), two previous deliveries, and or three or more previous deliveries). Maternal alcohol, heroin, cocaine, methadone, and tobacco use were collected as either used (yes) or no use (no) during pregnancy. Maternal comorbidities, including hypertensive disease in current pregnancy, gestational diabetes in current pregnancy, hepatitis B or C positive status, and women with history of prior preterm births were also extracted. Presence of these co-morbidities were recorded as ‘yes’ if present and ‘no’ if absent. Maternal HIV-RNA viral load and CD4 plasma levels were collected in the first, second and third trimesters of pregnancy. For the purposes of analyses, the number of mothers with HIV viral load <20 copies/mL during the first, second, and third trimesters of pregnancy were recorded. Other covariates extracted from maternal medical records include maternal age, and mode of delivery (based on viral load). Johns Hopkins Institutional Review Board (IRB) approval was received prior to data extraction.

### Statistical analysis

An assessment of normality of the data was done at the time of exploratory data analysis. Graphical and numerical methods, including the use of statistical tests, were used. The Kolmogorov-Smirnov test, as well as histograms, box plots, P-P plots and Q-Q plots were used to check our data for missing variables, assumptions, outliers and influential observations, and subsequently examined the relationships among explanatory covariates, and assess the direction and approximate size of relationships between explanatory and outcome covariates. TDF and TDF medication adherence, sociodemographic and clinical characteristics were compared, and their frequencies determined between the exposure’s groups (TDF versus TAF) as prespecified. Categorical variables were reported as proportions, and continuous measures were reported as medians and interquartile ranges. Differences between groups were assessed with Fisher’s exact tests and Chi-square test for categorical variables (as appropriate), and Mann-Whitney U tests for continuous variables. Logistic regression models were used to estimate the odds of medication adherence and its associated 95% confidence intervals, accounting for potential confounders of the association between TDF/TAF use and medication adherence. Statistical analyses were conducted in Stata, version 17.0 (StataCorp, College Station, TX; 2021).

## RESULTS

In the final cohort, sixty-six women received tenofovir disoproxil fumarate (TDF) while thirty-four women received tenofovir alafenamide (TAF). Baseline characteristics did not differ significantly between women who were on TDF compared to those who were on a TAF-based regimen, except for maternal age and CD4 counts in the first and third trimesters of pregnancy (Table 1). Pregnant women living with HIV on a TDF regimen were older than those in the TAF arm (median age of 32 years (interquartile range, IQR (29-36) in the TDF group and 29 years (IQR 25-32) in the TAF group; p value= 0.045). On the contrary, women on TAF-based regimen were more likely than those on a TDF regimen to have a higher CD4 count (median 470 cells/mm^3^ (IQR 355-594) in the TDF arm versus 669 cells/mm^3^ (514-750); p=0.035) in the third trimester of pregnancy. The majority of participants were Blacks [54/66 (82 %) in the TDF group and 31/34 (91%) in the TAF group, p value= 0.214)].

Medication adherence in the 1^st^ trimester (76% adherence to TDF versus 83% adherence to a TAF regimen; p=0.282), 2^nd^ trimester (82% adherence to TDF versus 88% adherence to a TAF regimen; p=0.924), and 3^rd^ trimesters (88% adherence to TDF versus 91% adherence to a TAF regimen; p=0.176) of pregnancy were higher in pregnant women living with HIV on TAF compared to those on TDF, but these differences in medication adherence were not statistically significant. Adherence to both medications increased from the first to the third trimesters of pregnancy (Table 1).

There were no significant differences between women on TDF and those on TAF with respect to maternal alcohol and cocaine use; heroin, methadone, and tobacco use; hypertension in pregnancy, preterm delivery, hepatitis B and C infections, and mode of delivery based on maternal viral load. Four women in the TDF arm (6%) were delivered by Cesarean for viral loads >1,000 copies/mL, while none was delivered in the TAF arm solely for a high viral load indication.

Table 2 shows the univariable and multivariable logistic regression analysis of the associations with medication adherence stratified by trimesters of pregnancy (first, second, and third trimesters of pregnancy). In the univariable and multivariable analysis in the first and second trimesters of pregnancy, there were no statistically significant associations between medication adherence and TDF or TAF use, parity, race/ethnicity, cocaine, tobacco, marijuana, and heroin use (Table 2). In the third trimester of pregnancy, multiparous women (parity of >=2) were more likely to be adherent to antiretroviral medications compared to nulliparous women. This finding was statistically significant in both the univariable (odds ratio, OR 1.31, 95% CI 1.12, 1.57; p<0.05) and multivariable (adjusted odds ratio, aOR 1.23, 95% CI 1.08, 1.52; p<0.05) analyses.

## DISCUSSION

This retrospective cohort study found that medication adherence levels to TDF and TAF are overall, high in pregnant women living with HIV. Although medication adherence was higher for women using TAF when compared to compare to TDF, the differences in adherence were not statistically significant. In the third trimester of pregnancy, multiparous women were more likely to be adherent to TDF/TAF antiretroviral medications compared to nulliparous women, a statistically significant finding.

Nulliparity was identified as a risk factor for poor antiretroviral medication adherence in this study. While it can be difficult to explain why adherence was better in multiparous women in this cohort of pregnant women living with HIV, a possible explanation why multiparous women had better adherence in our study is that nulliparous pregnant women sometimes forget to take their medications, partly because, as their first pregnancy, nulliparous pregnant women might be scared of taking medications, or due to adverse effects of the medications compared to the more experienced multiparous women who would take medications more consistently in their prior pregnancies.

Changes in medication adherence can occur at any time during pregnancy [23,24]. Although medication adherence generally increases as pregnancy increases from the first to the third trimesters of pregnancy [23], adherence can be modified by a woman’s’ medication dosing regimen complexity, pill burden, social support systems, among other predisposing factors [25]. While many pregnant women are motivated to increase medication adherence during pregnancy, keep their disease under control, and reduce the chances of perinatal transmission of HIV, the proportion of women who are poorly adherent to medications during pregnancy, especially in the first trimester of pregnancy, do so for fear and anxiety of potential harm to the fetus [23]. In this study, adherence to HIV medications increased from the first to the third trimester of pregnancy, consistent with findings from other studies [23].

There remains a critical, unmet need for objective, quantitative measures of adherence to antiretroviral medications among women living with HIV [19]. Non-pharmacological adherence measures such as patient self-report, pillbox checks and counts of pill days, and electronic apps are limited by response bias, as participants can respond to questions about adherence inaccurately, incompletely, or falsely. In addition, these subjective methods do not measure adherence directly [26,27]. Multiple studies have examined tenofovir diphosphate (TFV-DP), the active form of tenofovir in peripheral blood mononuclear cells (the site of action) as an objective measure of cumulative adherence [19,28,29]. Data from the MTN 001 trial [30] demonstrated that TFV-DP concentrations in peripheral blood mononuclear cells (PBMCs) could be used as objective measures of adherence [30]. While plasma tenofovir (TFV) did not correlate well with medication adherence in the MTN 001 trial, measurement of TFV-DP in PBMCs was an authentic and valid process of assessing TFV adherence [30]. Other methods to assess medication adherence include use of dried blood spots [31,32] and maternal hair [33]. Although TFV-DP in dried blood spots (DBS) can be used to measure accumulated drug, they represent drug exposures over 4-6 weeks (hair) or 6-8 weeks (DBS) [34,35] and might not efficiently capture current adherence patterns in pregnant women given the rapidly changing physiology of pregnancy. So, objective methods of adherence assessment, while better than subjective methods of adherence have limitations as well.

This study had some limitations. First, is the strict method in which we classified adherence (<100% versus 100% adherence). There have been less strict ways to classify medication adherence in literature (as <95% versus ≥95%; <90% versus ≥90%; or <80% versus ≥80%). However, despite our strict method of classifying adherence, there were no TDF or TAF groups in the first, second or third trimesters of pregnancy that were less than 75% adherent in this study. A second limitation of this study is that the comparatively small sample size of the study did not allow for sub-group analyses. With an increased sample size, it may have been possible to show other statistically significant differences in medication adherence patterns between women who used TDF compared to those who used TAF during pregnancy. Finally, although women subjectively reported medication adherence, it was difficult to objectively ascertain adherence to TDF/TAF medications in this study. This is important because non-pharmacologic adherence measures have limitations and may overestimate or underestimate TDF or TAF adherence [9,19].

The strengths of this study include the comparatively high number of pregnant women living with HIV managed at this single center - much more than the numbers currently managed at several other centers within the United States and other developed countries. Finally, because TAF use was part of routine obstetric care at this institution, the results of this study may be more generalizable to many obstetric practices within and outside the United States.

## CONCLUSION

Our results suggest that there are no differences in optimal adherence to TDF versus TAF based antiretroviral therapy in pregnant women living with HIV except for maternal parity. Utilizing objective methods of adherence are important, as objective methods of medication adherence are less prone to bias. Larger studies are required to describe intracellular PBMC TFV-DP concentrations, and establish TAF medication adherence benchmarks in pregnancy and postpartum in pregnant and postpartum women living with HIV.

## Figures and Tables

**Table 1.** Characteristics of Study Participants Stratified by Medication type (TDF versus TAF).

Variable	Tenofovir Disoproxil Fumarate (TDF) N=66	Tenofovir Alafenamide (TAF) N=34	P-value
**Medication adherence in the 1^st^ trimester**			
**<100 percent, n (%)**	16 (24)	6 (17)	0.282
**100 percent, n (%)**	50 (76)	28 (83)	
**Medical adherence in the 2^nd^ trimester**			
**<100 percent, n (%)**	12 (18)	4 (12)	0.924
**100 percent, n (%)**	54 (82)	32 (88)	
**Medication adherence in the 3^rd^ trimester**			
**<100 percent, n (%)**	8 (12)	3 (9)	0.176
**100 percent, n (%)**	58 (88)	31 (91)	
**Age (median in years; IQR)**	32 (29-36)	29 (25-32)	0.045
**Parity, n (%)**			
**Para 0**	9 (14)	9 (27)	0.246
**Para 1**	23 (35)	11 (32)	
**Para 2**	20 (30)	11 (32)	
**Para 3 or greater**	14 (21)	3 (9)	
**Race, n (%)**			
**Black/African American**	54 (82)	31 (91)	0.214
**Non-Black**	12 (18)	3 (9)	
**Number of mothers with viral load <20 copies/mL, n, (%)**			
**1^st^ trimester**	48 (73)	25 (74)	0.964
**2^nd^ trimester**	54 (82)	28 (82)	0.854
**3^rd^ trimester**	55 (84)	29 (86)	0.791
**CD4+ (cells/mm^3^), median, IQR**			
**1^st^ trimester**	569 (373-766)	667 (512-973)	0.050
**2^nd^ trimester**	444 (353-620)	510 (420-722)	0.230
**3^rd^ trimester**	470 (355-594)	669 (514-750)	0.035
**Alcohol use prior to pregnancy, n (%)**	1 (2)	2 (5)	0.980
**Cocaine use prior to pregnancy, n (%)**	5 (8)	0 (0)	0.100
**Heroin use prior to pregnancy, n (%)**	2 (3)	1 (3)	0.980
**Methadone use in pregnancy, n (%)**	3 (5)	2 (6)	0.771
**Tobacco use prior to pregnancy, n (%)**	6 (9)	7 (20)	0.105
**Hypertension during pregnancy, n (%)**	5 (8)	3 (9)	0.127
**Gestational diabetes mellitus, n (%)**	2 (3)	2 (6)	0.201
**Preterm delivery, n (%)**	9 (14)	3 (9)	0.496
**Hepatitis B, n (%)**	1 (2)	1 (3)	0.629
**Hepatitis C, n (%)**	3 (5)	0 (0)	0.207
**Mode of delivery (n (%)**			
**Vaginal delivery**	41 (62)	17 (50)	0.212
**Cesarean delivery**	25 (38)	17 (50)	
**Cesarean delivery for viral load, n (%)**	4 (6)	0 (0)	0.083

**Table 2.** Parameter estimates and 95% confidence intervals of the crude and adjusted hazard ratios for associations with adherence using logistic regression.

Variable	Unadjusted (crude) OR (95% CI)	Adjusted OR (95% CI) *
**First Trimester Medication Adherence**		
**Antiretroviral**		
**TDF**	Referent	Referent
**TAF**	2.02 (0.55 - 7.37)	2.35 (0.52 - 10.47)
**Parity**		
**1**	Referent	Referent
**2 or more**	0.99 (0.73 - 1.35)	1.00 (0.72 - 1.40)
**Race**		
**Non-Black**	Referent	Referent
**Black**	1.15 (0.27 - 5.00)	0.84 (0.16 - 4.54)
**Cocaine use**	0.13 (0.01 - 1.56)	0.25 (0.01 - 6.87)
**Tobacco use**	0.34 (0.67 - 1.75)	0.14 (0.06 - 3.10)
**Marijuana use**	0.54 (0.12 - 2.47)	0.43 (0.08 - 2.45)
**Heroin use**	0.13 (0.01 - 1.56)	0.59 (0.18 - 19.5)
**Second Trimester Medication Adherence**		
**Antiretroviral**		
**TDF**	Referent	Referent
**TAF**	1.09 (0.17 - 7.00)	0.92 (0.09 - 9.46)
**Parity**		
**1**	Referent	Referent
**2 or more**	0.96 (0.56 - 1.65)	0.90 (0.46 - 1.75)
**Race**		
**Non-Black**	Referent	Referent
**Black**	3.60 (0.53 - 24.4)	3.40 (0.29 - 39.8)
**Cocaine use**	0.06 (0.03 - 1.21)	0.07 (0.01 - 4.99)
**Tobacco use**	0.18 (0.03 - 1.30)	0.41 (0.22 - 7.41)
**Marijuana use**	0.25 (0.04 - 1.68)	0.17 (0.02 - 1.79)
**Heroin use**	0.06 (0.03 - 1.21)	0.97 (0.01 - 92.1)
**Third Trimester Medication Adherence**		
**Antiretroviral**		
**TDF**	Referent	Referent
**TAF**	1.03 (0.99 - 1.07)	0.86 (0.66 - 1.08)
**Parity**		
**1**	Referent	Referent
**2 or more**	1.31 (1.12 - 1.57) **	1.23 (1.08 - 1.52) **
**Race**		
**Non-Black**	Referent	Referent
**Black**	0.79 (0.52 - 1.27)	0.83 (0.41 - 1.48)
**Cocaine use**	0.34 (0.03 - 3.87)	0.24 (0.01 - 3.57)
**Tobacco use**	0.62 (0.06 - 7.07)	0.58 (0.05 - 6.68)
**Marijuana use**	0.19 (0.01 - 2.33)	0.14 (0.03 - 1.89)
**Heroin use**	0.93 (0.74 - 1.39)	0.87 (0.59 - 1.07)
